# Non-lactose fermenting *Escherichia coli*: Following in the footsteps of lactose fermenting *E. coli* high-risk clones

**DOI:** 10.3389/fmicb.2022.1027494

**Published:** 2022-11-03

**Authors:** Razib Mazumder, Arif Hussain, Jody E. Phelan, Susana Campino, S. M. Arefeen Haider, Araf Mahmud, Dilruba Ahmed, Md Asadulghani, Taane G. Clark, Dinesh Mondal

**Affiliations:** ^1^Laboratory Sciences and Services Division, International Centre for Diarrhoeal Disease Research, Bangladesh (icddr,b), Dhaka, Bangladesh; ^2^Department of Infection Biology, London School of Hygiene and Tropical Medicine, London, United Kingdom; ^3^Clinical Microbiology and Immunology Laboratory, Laboratory Sciences and Services Division, International Centre for Diarrhoeal Disease Research, Bangladesh (icddr,b), Dhaka, Bangladesh; ^4^Biosafety and BSL3 Laboratory, Biosafety Office, International Centre for Diarrhoeal Disease Research, Bangladesh (icddr,b), Dhaka, Bangladesh

**Keywords:** genomic epidemiology, high risk clone, carbapenem resistance, ESBL – *Escherichia coli*, ST131 and non-ST131 lineages, non lactose fermenter

## Abstract

Multi-resistant pathogenic strains of non-lactose fermenting *Escherichia coli* (NLF *E. coli*) are responsible for various intestinal and extraintestinal infections. Although several studies have characterised such strains using conventional methods, they have not been comprehensively studied at the genomic level. To address this gap, we used whole-genome sequencing (WGS) coupled with detailed microbiological and biochemical testing to investigate 17 NLF *E. coli* from a diagnostic centre (icddr,b) in Dhaka, Bangladesh. The prevalence of NLF *E. coli* was 10%, of which 47% (8/17) exhibited multi-drug resistant (MDR) phenotypes. All isolates (17/17) were confirmed as *E. coli* and could not ferment lactose sugar. WGS data analysis revealed international high-risk clonal lineages. The most prevalent sequence types (STs) were ST131 (23%), ST1193 (18%), ST12 (18%), ST501 (12%), ST167 (6%), ST73 (6%) and ST12 (6%). Phylogenetic analysis corroborated a striking clonal population amongst the studied NLF *E. coli* isolates. The predominant phylogroup detected was B2 (65%). The *bla*_CTX-M-15_ extended-spectrum beta-lactamase gene was present in 53% of isolates (9/17), whilst 64.7% (11/17) isolates were affiliated with pathogenic pathotypes. All extraintestinal pathogenic *E. coli* pathotypes demonstrated β-hemolysis. Our study underscores the presence of critical pathogens and MDR clones amongst non-lactose fermenting *E. coli.* We suggest that non-lactose fermenting *E. coli* be considered equally capable as lactose fermenting forms in causing intestinal and extraintestinal infections. Further, there is a need to undertake systematic, unbiased monitoring of predominant lineages amongst non-lactose fermenting *E. coli* that would help in better treatment and prevention strategies.

## Introduction

*Escherichia coli* is a versatile gram-negative bacterium with huge genetic diversity (Nia Santos Braz et al., 2020). It is the most common organism responsible for opportunistic infections. Its primary habitat includes the lower intestinal tract of humans and animals (Nia Santos Braz et al., 2020). However, infections with variant strains of *E. coli* are responsible for various clinical manifestations ranging from diarrhoea, urinary tract infections, and life-threatening septicemia, resulting in over 2 million deaths every year globally (Hussain et al., 2012; Huang et al., 2021). Moreover, these infections are often associated with cephalosporin and carbapenem-resistant strains, impacting the mortality of patients and imparting huge healthcare costs (Logan and Weinstein, 2017).

*Escherichia coli* are non-pathogenic facultative anaerobic flora of the intestinal tract in humans. The gram-negative *E. coli* bacilli ferments lactose to produce hydrogen sulfide. However, up to 20% of *E. coli* isolates from patients are reported to be atypical, which are slow or non-lactose fermenters due to the deficiency in enzyme lactose permease encoded by the *lacY* gene. (Nicoletti et al., 1988; Hossain, 2012; Chang et al., 2014; Yaratha et al., 2017; Johnson et al., 2019). Non-lactose fermenting (NLF) *E. coli* can be identified as colourless, transparent colonies on MacConkey agar and by negative lactose (sugar) fermentation tests (Hossain, 2012; Siqueira et al., 2021). Various strains of *E. coli* are equipped to cause different forms of enteric and extraintestinal infections in human hosts with varying propensities (Kaper et al., 2004). The pathogenic strains of *E. coli* belong to both lactose fermenting and non-lactose fermenting *E. coli* types. However, little attention is given to these atypical strains of *E. coli,* particularly the non-lactose fermenting (NLF) *E. coli* in routine diagnostic testing laboratories.

*Escherichia coli* is a well-established etiological agent of diarrhoea (Colonna et al., 1992; Nataro and Kaper, 1998; Hossain, 2012). Diarrheagenic strains of *E. coli* fall into different categories possessing distinct pathogenic mechanisms, the three important *E. coli* strains include enteropathogenic *E. coli* (EPEC), enterohemorrhagic *E. coli* (EHEC), and enteroaggregative *E. coli* (EAEC) (Nataro and Kaper, 1998). These *E. coli* categories cause a range of clinical syndromes such as watery diarrhoea in children, persistent diarrhoea, traveller’s diarrhoea and hemolytic uremic syndrome (Nataro and Kaper, 1998). A few studies have reported that NLF *E. coli* strains were identified as typical pathogenic *E. coli* isolates, having a possible diarrheagenic role (Nicoletti et al., 1988; Colonna et al., 1992; Hossain, 2012).

Urinary tract infections (UTIs) represent one of the most common bacterial infections. It is a serious public health problem affecting around 150 million people worldwide (Flores-Mireles et al., 2015). *E. coli* is the most common pathogen responsible for (un)complicated urinary tract infections (Flores-Mireles et al., 2015). Such strains harbour multiple virulence factors, which play their role in the pathophysiology of UTIs. These virulence factors affect bacterial colonisation and evasion of host defences (Hussain et al., 2014; Mazumder et al., 2020a). Recent reports suggest that clonal groups of multi-resistant, multi-virulent *E. coli* are increasingly responsible for UTIs, presenting a challenge for their treatment (Riley, 2014). In recent years, a few studies have demonstrated that NLF *E. coli* have been increasingly isolated from urine specimens in microbiology laboratories and have reported their clinical significance concerning antibiotic resistance, virulence and emerging clones (Chang et al., 2014; Chakraborty et al., 2016; Wu et al., 2017).

Rapid identification of bacterial pathogens in microbiology laboratories is critical for initiating successful infection treatment. Screening of gram-negative bacteria from urine and stool samples is routinely performed on MacConkey agar, and the colourless transparent NLF *E. coli* variants are usually missed in the screening process as little attention is given to these atypical strains of *E. coli*. Although reports of such isolates are limited worldwide, a few studies have reported the occurrence and characterisation of NLF *E. coli* from clinical specimens. They have characterised the strains using only conventional methods. This study aims to decipher the biochemical profiles, population structure, and genomic characteristics of NLF *E. coli* from stool and urine samples isolated at a referral diagnostic centre (International Center for Diarrheal Disease Research, Bangladesh; icddr,b) in Dhaka, Bangladesh. Studies such as these will inform us about the evolutionary trajectories of atypical *E. coli* variants and shed light on their clinical and public health significance.

## Materials and methods

### Bacterial isolates and their biochemical and serological characterisation

A total of 175 *E. coli* isolates were sourced from urine (*n* = 98; 56%) and stool (*n* = 77; 44%) culture plates. The isolates were collected randomly from the Clinical Microbiology Laboratory of the icddr,b, based in Dhaka, Bangladesh. The isolates were sampled between 2019 and 2020 as part of a larger 1% AMR surveillance study. From this collection of 98 urine and 77 stool *E. coli* isolates, we identified 9 (9.2%) and 8 (10.4%) NLF *E. coli* isolates, respectively. These 17 NLF *E. coli* were subjected to complete biochemical profiling and whole-genome sequencing (WGS) using an Illumina platform (NextSeq 500). NLF *E. coli* were presumptively identified by their inability to ferment lactose on the MacConkey agar. The presence of *Shigella* spp. was ruled out by serotyping using slide agglutination test employing a commercial *Shigella* grouping antisera kit that includes group A (*S. dysenteriae*), B (*S. flexineri*), C (*S. boydii*) and group D (*S. sonnei*) antisera from Denka Seiken Co. Ltd. Tokyo, Japan. Further, confirmation was made by standard biochemical methods that include motility, indole and urease tests (using MIU agar), triple sugar iron (TSI) agar test to visualise glucose and lactose fermentation reactions, catalase test (using 3% H_2_O_2_), oxidase test (using 1% aqueous solution of tetra-methyl-p-phenylenediamine dihydrochloride), citrate utilisation test (using Simmons citrate agar) and acetate utilisation test (using Acetate agar) (Hawkey, 2006; Aditi et al., 2017). As mentioned in Table 1, fermentation of other sugars was confirmed using carbohydrate broth with bromothymol blue as a pH indicator, employing previously described methods (Bopp et al., 2003). Voges-Proskauer test was performed in MRVP broth and the reaction was visualised by adding 5% α naphthol and 40% potassium hydroxide (KOH) (Hawkey, 2006). Gelatine liquefaction and ONPG tests were performed as described elsewhere (Hawkey, 2006; Nusrat et al., 2017). Decarboxylation reaction of amino acids was performed on decarboxylase broth supplemented by 1% of the appropriate amino acids as described earlier (Brooks and Sodeman, 1974). The Analytical profile index was evaluated using the API 20E kit (bioMérieux) as per the manufacturer’s instructions (Table 1). Hemolytic properties of strains were evaluated using 5% sheep blood agar plates. Clearing zones around the colonies were suggestive of beta-haemolysis.

**Table 1:** Microbiological and biochemical characteristics of 17 NLF *E. coli* isolates.

Sl. no.	Biochemical tests	No. (%)	
		Positive	Negative
1	Catalase test	17 (100)	0
2	Oxidase test	0	17 (100)
3	TSI agar		
	a. Acid production in slant	17 (100)	0
	b. Acid production in butt	17 (100)	0
	c. Hydrogen sulfide production(H_2_S)	0	17 (100)
	d. Gas production	17 (100)	0
4	Motility Indole Urease Test (MIU)		
	a. Motility	17 (100)	0
	b. Indole Production	17 (100)	0
	c. Urea hydrolysis	0	17 (100)
5	Simmons Citrate reaction test	0	17 (100)
6	Acetate	17 (100)	0
7	Sugar fermentation		
	a. Glucose	17 (100)	0
	b. Lactose	0	17 (100)
	c. Sucrose	11 (65)	6(35)
	e. Mannose	17 (100)	0
	f. Arabinose	17 (100)	0
	g. Sorbitol	17 (100)	0
	h. Mannitol	17 (100)	0
	i. Inositol	0	17 (100)
8	Ortho-Nitrophenyl-β-galactoside (ONPG)	17 (100)	0
9	Gelatine liquefaction	0	17 (100)
10	Vogas-proskauer	0	17 (100)
11	Lysine decarboxylase	17 (100)	0
12	Ornithine decarboxylase	14(82)	3 (18)
13	Arginine Dihydrolase	11 (65)	6(35)
API 20E Results [API profile: isolates]			
1	API profile: 7144532	A3, A4, A5, A7, U1, U7, U8, U12, U13, U14, U15	
2	API profile: 5144572	A6, U4, U6	
3	API profile: 5044552	A1, A8, A9	
Bacteriological Characteristics [17 (100%)]			
1	Growth on MacConkey Agar	Colourless and transparent (NLF colony)	
2	Growth on SS agar	Colourless and transparent (NLF colony)	
3	Growth on CHROMagar™ Orientation	Pink colour colony	
4	Growth Temperature	(26–42°C)	
5	Gram’s staining	Gram-negative rods	

### Antimicrobial susceptibility testing

The Genome Centre at icddr,b reassessed 17 NLF *E. coli* isolates with an extensive panel of 18 antibiotics (Oxoid, US) by the Kirby-Bauer disc diffusion method. The results were interpreted using the 2019 Clinical and Laboratory Standards Institute (CLSI) guidelines. Upon antibiotic susceptibility testing, the NLF *E. coli* isolates identified as intermediate and resistant were considered as resistant. They were classified as multi-drug resistant (MDR) if they were non-susceptible to at least one agent belonging to three different antimicrobial classes (Magiorakos et al., 2012).

### Whole-genome sequencing

Total bacterial DNA was isolated and purified from the overnight grown bacterial cultures using the QIAmp DNA Mini kit (Qiagen, Germany). DNA QC and quantification were performed employing a Nanopore spectrophotometer (Thermo Fisher Scientific, United States) and Qubit 4.0 fluorometer (Life Technologies; Baddam et al., 2020). DNA libraries for the short-read paired-end sequencing were prepared using the Nextera DNA Flex library prep kit (Illumina). Size selected and pooled libraries were sequenced at the icddr,b Genome Centre in Illumina NextSeq 500 system using a NextSeq v2.5 reagent kit (2 × 150 bp) (Mazumder et al., 2020b).

### Sequence assembly and annotation

High-quality reads were obtained by filtering the paired-end data using fastp (Chen et al., 2018). *De novo* assemblies were generated using SPAdes (v.3.11.11) (Bankevich et al., 2012). NCBI Prokaryotic Genome Annotation Pipeline (PGAP) (Tatusova et al., 2016) was used for annotating the genome assemblies. The genome statistics from the resulting file were gleaned using the QUAST software (v5.2.0) (Gurevich et al., 2013).

### *In silico* analysis

Species identification was confirmed using Kmer Finder software (v3.2) (Hasman et al., 2014; Larsen et al., 2014; Clausen et al., 2018). Phylogenetic groups of the genomes were inferred using the Clermon Typing tool (Beghain et al., 2018). The sequence types (STs) were determined by submitting the contigs to MLST (v2.0; scheme #1) (Larsen et al., 2012). SerotypeFinder 2.0 (Joensen et al., 2015) and CHTyper 1.0 (Roer et al., 2018) software tools were used for elucidating serotypes and clonotypes, respectively.

Resistance genes were screened by BLASTn analysis of the genome assemblies against the data downloaded from the ResFinder database (Bortolaia et al., 2020). The 70% query coverage and 90% identity indicated a positive hit in the genome. Plasmid incompatibility groups were determined by PlasmidFinder (v2.1) (Carattoli et al., 2014). Mobile genetic elements associated with acquired antimicrobial resistance (AMR) genes were determined using Mobile Element finder v1.0.3 (Johansson et al., 2021). Chromosomal point mutations conferring fluoroquinolone resistance were identified by PointFinder software (Zankari et al., 2017). Contigs with *bla*_NDM-5_ and *bla*_CTX-M-15_ were analysed by BLASTn analysis against the database in NCBI to identify the chromosomal or plasmid origin of these genes.

Virulence gene content of strains was determined by BLASTn analysis of 17 genomes against the *E. coli* database selected in the virulence factor database (VFDB; Chen et al., 2005). The genes with 90% identity and 70% query coverage were considered present. Isolates were classified as extraintestinal pathogenic *E. coli* based on Johnson’s criteria (Johnson and Stell, 2000). The *in-silico* analysis described above was performed on default parameters unless otherwise stated.

### Phylogenomic and pangenomic analysis

The phylogenetic tree was inferred with the reference sequence alignment-based phylogeny builder (REALPHY) software (Bertels et al., 2014). *E. coli* K-12 MG1655 genome (accession no.U00096) was used as the single reference sequence and PhyML (Guindon et al., 2010) was used as the treebuilder. The phylogenetic tree was later visualised using the Interactive Tree of Life (iTOL; Letunic and Bork, 2016). Pan-genome analysis was conducted using the Anvi’o (v7.1) software package (Eren et al., 2015) following the microbial pan-genomics analysis workflow.

## Results

### Bacterial and biochemical characteristics

The colourless NLF colonies on the MacConkey agar plate appeared like *Shigella* spp. colonies. Colonies were colourless even in 72-h old MacConkey agar cultures, removing the possibility of late lactose fermenters. Serology of all colonies revealed negative reactions to the four serogroups of *Shigella: S. flexneri, S. dysenteriae, S. boydii, and S. sonnei*. Further, streaking of colonies on CHROMagar Orientation media revealed small, pink-red colonies typical of *E. coli*. The isolates were then tested by routine biochemical tests as described in Table 1 and identified as *E. coli*. Notably, all isolates were O-nitrophenyl-beta-D-galactopyranoside positive and did not ferment lactose sugar. Further confirmation was done by generating a biochemical profile for each isolate using 20 reactions of the API 20E strip. The API results gave three distinct profiles: 7144532 (*n* = 11), 5,044,552 (*n* = 3) and 5,144,572 (*n* = 3) and identified all isolates as *E. coli* with 99.8% probability (Table 1). Gram staining confirmed the presence of gram-negative bacilli. The optimum growth temperate of NLF *E. coli* isolates ranged from 26° to 42°C.

### Molecular analysis and phylogenomics of NLF *Escherichia coli* isolates

WGS of the 17 NLF *E. coli* study isolates yielded an average genome size of 5,160,336 bp (range 4,890,228–5,480,336) and the average GC content was 50.6% (range 50.3–50.7%). KmerFinder software results confirmed all the isolates as *E. coli*. On average, the genome assemblies had a genome coverage of 86-fold (range: 62–124-fold; Table 2).

**Table 2:** Genomic features of the 17 whole-genome sequenced NLF *E. coli* isolates.

Sl no.	Isolate ID	Strain ID	Source	Isolation year	Genome coverage (X)	Contig no. (>500 bp)	Genome size (bp)	No. CDS	GC %	Accession no.
1	A1	BDEc_NLF-A1	Stool	2019	91	106	5,305,815	5,077	50.5	JAMKCS000000000
2	A3	BDEc_NLF-A3	Stool	2019	101	103	5,480,336	5,335	50.6	JAMKCT000000000
3	A4	BDEc_NLF-A4	Stool	2019	96	96	5,019,286	4,772	50.7	JAMKCU000000000
4	A5	BDEc_NLF-A5	Stool	2019	63	77	5,074,445	4,830	50.5	JAMKCV000000000
5	A6	BDEc_NLF-A6	Stool	2019	121	84	4,890,228	4,656	50.6	JAMKCW000000000
6	A7	BDEc_NLF-A7	Stool	2020	90	70	4,978,296	4,710	50.7	JAMKCX000000000
7	A8	BDEc_NLF-A8	Stool	2020	124	151	5,033,902	4,934	50.6	JAMKCY000000000
8	A9	BDEc_NLF-A9	Stool	2020	91	43	5,068,010	4,850	50.6	JAMKCZ000000000
9	U-1	BDEc_NLF-U1	Urine	2019	71	44	5,073,930	4,893	50.6	JAMKDA000000000
10	U-4	BDEc_NLF-U4	Urine	2019	62	53	5,251,806	4,988	50.4	JAMKDF000000000
11	U-6	BDEc_NLF-U6	Urine	2019	79	44	5,054,491	4,847	50.6	JAMKDG000000000
12	U-7	BDEc_NLF-U7	Urine	2019	88	109	5,302,191	5,061	50.5	JAMKDH000000000
13	U-8	BDEc_NLF-U8	Urine	2020	71	56	5,186,272	5,002	50.6	JAMKDI000000000
14	U-12	BDEc_NLF-U12	Urine	2020	83	57	5,021,789	4,793	50.7	JAMKDB000000000
15	U-13	BDEc_NLF-U13	Urine	2020	84	69	5,262,816	5,008	50.5	JAMKDC000000000
16	U-14	BDEc_NLF-U14	Urine	2020	70	145	5,304,507	5,153	50.7	JAMKDD000000000
17	U-15	BDEc_NLF-U15	Urine	2020	75	79	5,417,595	5,190	50.36	JAMKDE000000000

**Figure 1:**
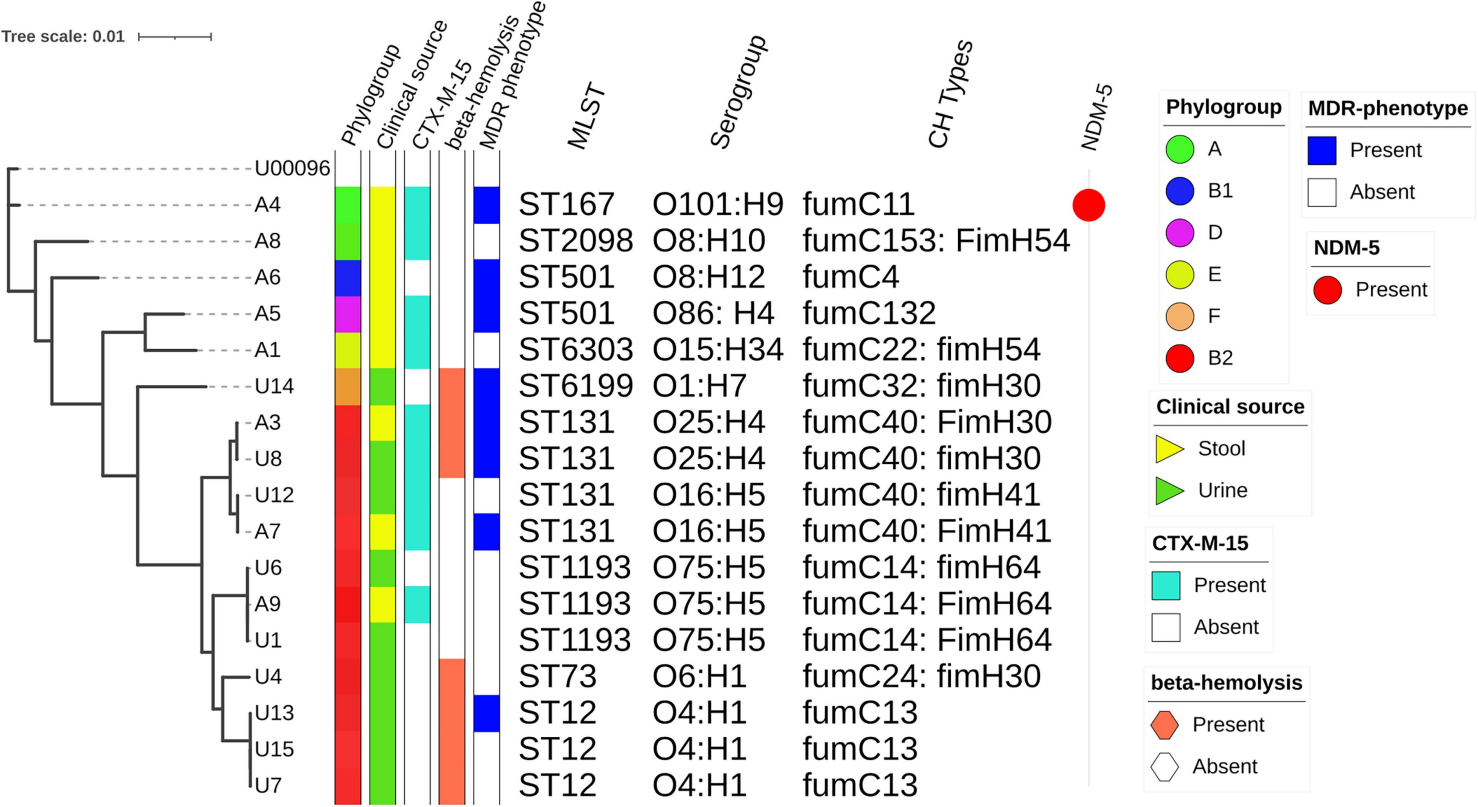
Phylogenetic relationships amongst sequenced NLF *E. coli* genomes. The maximum likelihood phylogenetic tree is based on the alignment of detected core genome SNPs of 17 NLF *E. coli* genomes with the MG1655 genome strain as a reference genome. The phylogroups, clinical source, *bla*_CTX-M-15_ status, beta-hemolysis, MDR status, STs, serogroups, CH types and *bla*_NDM-5_ status are shown to the right of the tree.

According to *in silico* MLST, isolates were classified into nine distinct sequence types (STs) and they represented some of the international high-risk lineages (Figure 1). ST131 was the predominant ST that accounted for 4 (23%) of the 17 NLF *E. coli* isolates. ST1193 and ST12 were present in equal proportions, 3 (18%) each, followed by two ST501 strains (12%). Other significant STs detected were ST167 (6%), ST73 (6%) and ST12 (6%). The predominant phylogroup detected was B2 (65%) which comprised all isolates affiliated with ST131, ST1193, ST12 and ST73. This was followed by group A (*n* = 2: 1ST167; 1 ST2089); other phylogroups were represented by single isolates (Figure 1). By serogroup, the 4 ST131 isolates were either O16: H4 (*n* = 2: fumC40: fimH41) or O25: H4 (*n* = 2: fumC40: fimH30; Figure 1). All ST1193 isolates were O75: H5 with fumC14: fimH64. All ST12 isolates were O4: H1and had fumC13. Other STs exhibited diverse serotypes and CH types (Figure 1).

Based on the alignment of detected core genome SNPs, a phylogenetic tree was built for 17 NLF *E. coli* from our study with the MG1655 genome as the reference genome. There was relatively low diversity of the studied NLF *E. coli* as most strains clustered together, resulting in clades and subclades. The analysis clustered the 17 isolates into two major clades, one tight cluster corresponding to phylogroup B2 and the other loosely clustered clade included strains from A, B1, D and F phylogroup. Compared to NLF *E. coli* from stool, the urine *E. coli* isolates formed a tight clade with strains belonging to ST12, ST1193 and ST131. Three strains from stool origin clustered closely with urine strains reinforcing the gut as a possible reservoir of UTI-causing agents. The *bla*_CTX-M-15_, MDR phenotype and β-hemolysis did not correspond with the clusters indicating their widespread presence across *E. coli* lineages. However, the sequence types, serogroups, and CH-types corresponded closely with the phylogenetic clusters of *E. coli* genomes. The ST131 strains with O16: H5 (fumC40: fimH41) formed a subcluster with ST131 strains having O25: H4 (fumc40: fimH30), indicating there are sub-lineages within ST131.

**Figure 2:**
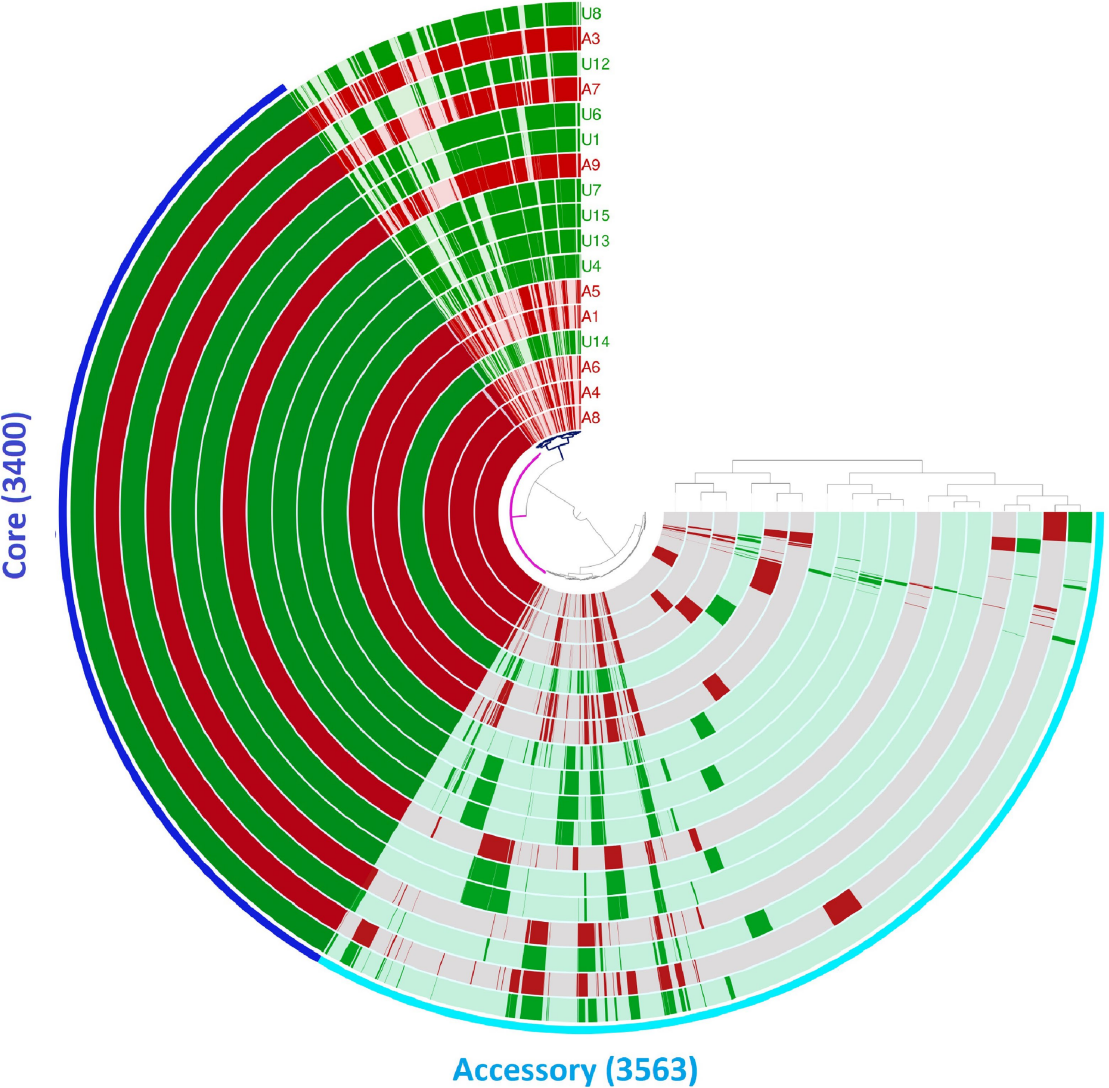
Pan-genome analysis of 17 NLF *E. coli* genomes from Anvi’o pan-genomics suite. The circles represent individual genomes arranged as per their pan-genomic phylogenetic relationship depicted by the outside dendrogram. In the circles, dark colour indicates the presence of a gene group and light colour its absence.

We also analysed the pan-genomes of the 17 NLF *E. coli* genomes (Figure 2). The number of core and accessory genes identified was 3,400 and 30,563, respectively. Contrary to the core-genome-based phylogenetic tree, no two genomes shared the same gene content with respect to the pan-genome. A closer look into the pan-genome dendrogram reflects a clustering pattern that mimics the clades of the core genome-based phylogenetic tree (Figure 2). These observations indicate that the 17 NLF *E. coli* genomes do not differ drastically, even regarding their accessory gene content. They all have a few unique gene families (Figure 2).

### Virulence genes

**Figure 3:**
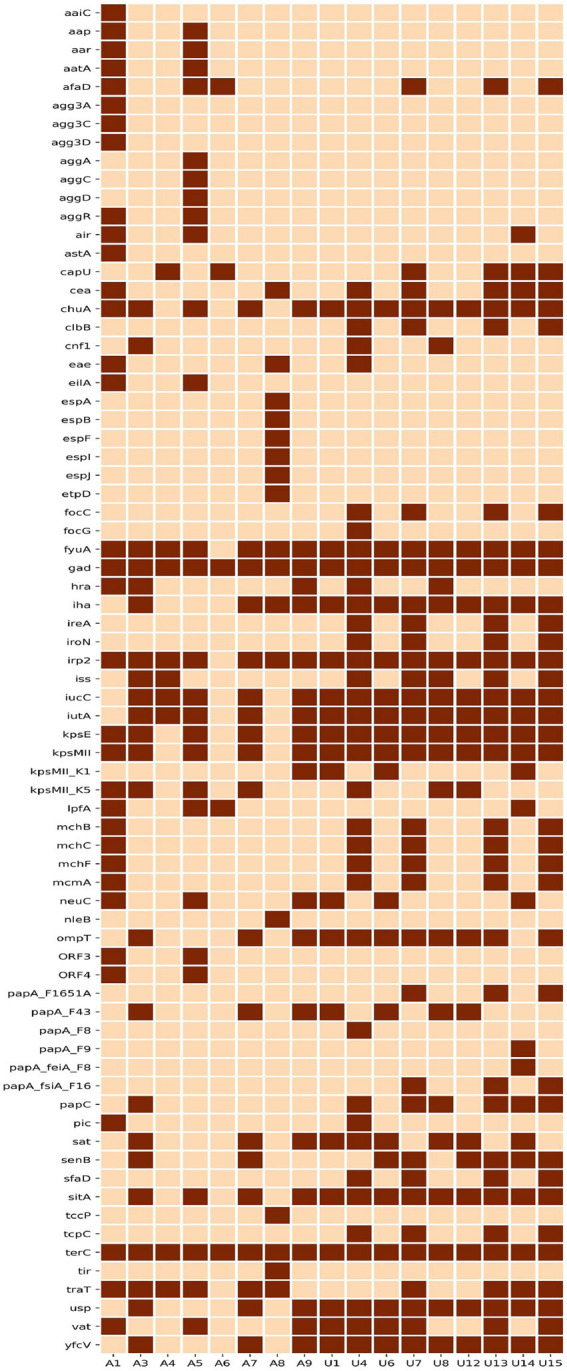
Heat map depicting the distribution of 73 virulence genes amongst 17 NLF *E. coli* genomes. Dark brown represents the presence, and light brown blocks represent a virulence gene’s absence.

Our analysis identified 73 virulence-associated genes out of 335 genes analysed. The distribution of 73 virulence genes amongst 17 NLF *E. coli* isolates is shown in Figure 3. Overall, 65% (11/17) of all NLF *E. coli* isolates were assigned to different pathotypes, as classified by the presence of specific virulence markers. Most urine NLF *E. coli* isolates were assigned to the extraintestinal pathogenic pathotype (67%, 6/9). Suspected diarrheagenic variants comprised 62% (5/8) of the stool NLF *E. coli* isolates. The most prevalent pathotype amongst diarrheagenic variants was enteroaggregative *E. coli* (38%), which harboured both *aggR* and *aatA* genes, followed by enteropathogenic *E. coli* (13%) that showed the presence of intimin gene (*eae*). One isolate from stool origin was also classified as extraintestinal pathogenic *E. coli*. The remaining six isolates (6/17) that did not belong to any pathotype included three strains from ST1193, two from ST131 and one from ST501. Out of four ST131 studied strains, two O25: H4 serogroup (fimH30) strains qualified as extraintestinal pathogenic and two strains with O16: H5 serogroup (fimH41) did not qualify as extraintestinal pathogenic. Amongst all the pathotypes, the seven (100%) ExpEC strains produced β-hemolysis in 5% sheep blood agar. This hemolytic phenotype was strongly associated with alpha-hemolysin genes (*hlyA*, *hlyB* and *hlyD*). Although the three ST1193 isolates did not qualify as extraintestinal pathogenic *E. coli* pathotypes, they all carried a similar set of virulence genes, including adhesins (*fimH*), toxins (*sat, hlyE/clyA*), siderophores (*chuA, fyuA, sitABCD, iutA*) and others. The NLF *E. coli* sourced from urine had a higher prevalence of virulence genes, with higher aggregate virulence scores (median 90; range 72–114) compared to those of stool NLF *E. coli* isolates (median 73; range 62–116).

### Antimicrobial resistance

**Figure 4:**
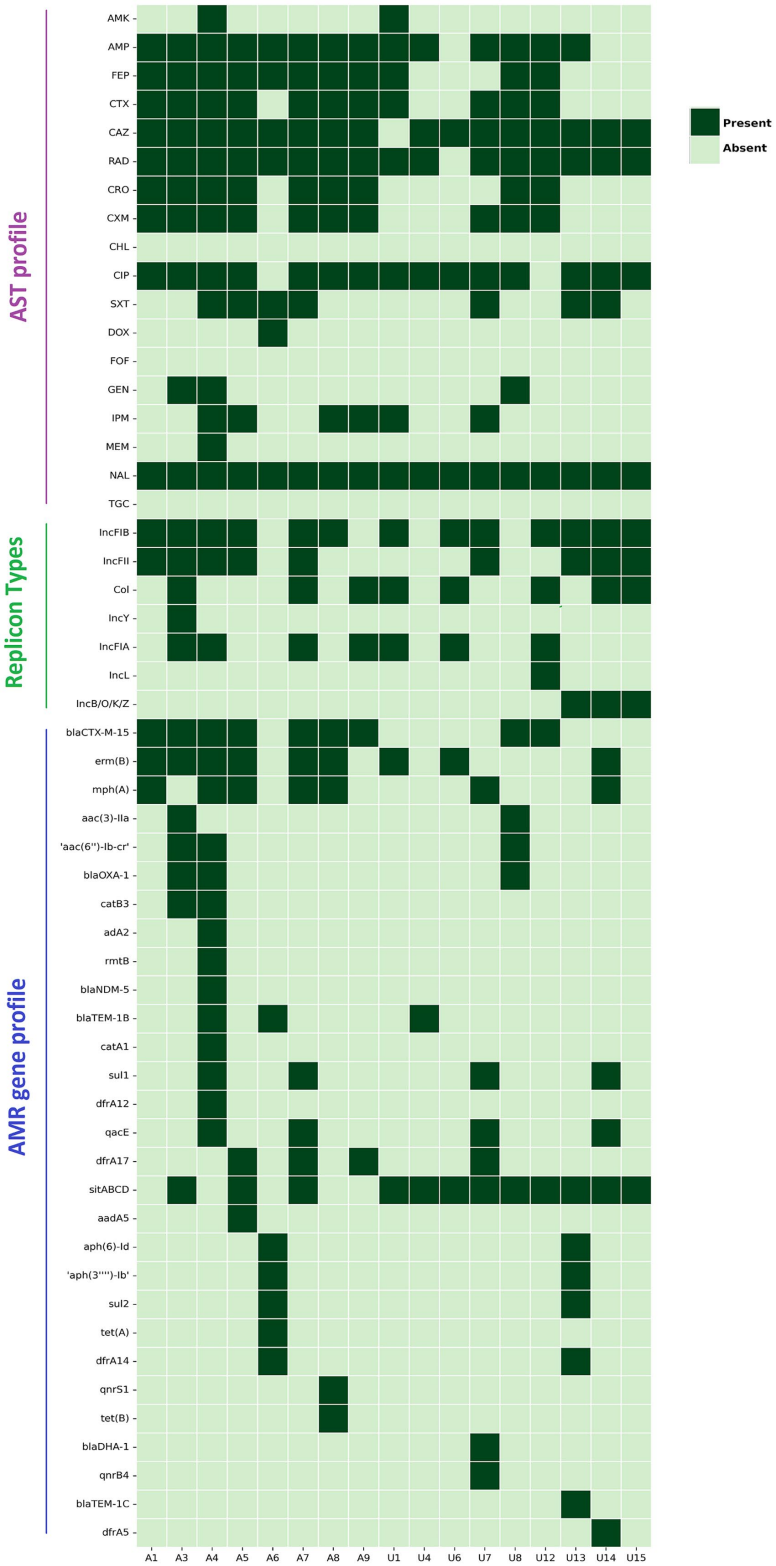
Heat map demonstrating the AST profile, plasmid replicon types and acquired AMR gene profile of 17 NLF *E. coli* isolates/genomes. Dark green represents resistance/presence, and light green blocks represent the sensitivity/absence of a particular trait. Definition of abbreviated antibiotics for AST: AMK, amikacin; Amp, ampicillin; FEP, cefepime; CTX, cefotaxime; CAZ, ceftazidime; RAD, cephradine; CRO, ceftriaxone; CXM, cefuroxime; CHL, chloramphenicol; CIP, ciprofloxacin; SXT, trimethoprim-sulfamethoxazole; DOX, doxycycline; FOF, fosfomycin; GEN, gentamicin; IPM, imipenem; MEM, meropenem; NAL, nalidixic acid; TGC, tigecycline.

The phenotypic and genotypic trends of AMR of the studied NLF *E. coli* strains were investigated (Figure 4). Overall, the NLF *E. coli* strains from the stool and urine samples showed high antimicrobial drug resistance rates to nalidixic acid (100%), ceftazidime (94%), cefradine (94%), followed by ciprofloxacin (88%), ampicillin (82%), cefotaxime (65%) and cefepime (65%). The resistance rates to cefuroxime (59%), ceftriaxone (53%), co-trimoxazole (41%), imipenem (35%) were moderate and the resistance rates to gentamicin (18%), amikacin (12%), doxycycline (6%), meropenem (6%) were low. In contrast, there appeared to be no resistance to chloramphenicol, fosfomycin and tigecycline. Compared to the stool isolates, the urine NLF *E. coli* isolates had relatively low resistance to amikacin, cefepime, ceftriaxone and cefuroxime; consequently, there were 63% (5/8) MDR isolates in the stool category and 33% (3/9) MDR isolates in urine category.

We detected 31 unique AMR gene alleles belonging to different classes, with most genomes exhibiting at least 6 AMR genes (Figure 4). The AMR genes had a relatively similar distribution between the stool and urine NLF *E. coli* isolates, with strains from both the groups having a minimum of 6 AMR genes per genome, albeit with variation amongst isolates (*E. coli* range: stool 3–15; urine 2–13). No specific AMR profile was associated with isolates from a particular clinical source.

A variety of ESBL gene variants encoded cephalosporin resistance. Amongst the identified ESBL genes in NLF *E. coli* isolates, *bla*_CTX-M-15_, was predominant (9/17, 53%), but we also identified *bla*_OXA-1_, *bla*_TEM1B_, *bla*_DHA-1_ and *bla*_TEM-1C_ genes in several isolates (Figure 4). We observed perfect concordance (100%) between the presence of *bla*_CTX-M-15_ gene and resistance phenotypes to the following cephalosporin antibiotics, cefradine, cefotaxime, ceftazidime, ceftriaxone, cefuroxime and cefepime. One *bla*_CTX-M-15_ positive stool NLF *E. coli* harboured a *bla*_NDM-1_ gene; this strain (A4) demonstrated difficult-to-treat resistance (DTR) as it was resistant to 14 of 18 antibiotics tested, including all first-line agents. It was sensitive to chloramphenicol, doxycycline, fosfomycin and tigecycline. The probable genome locus of the *bla*_CTX-M-15_ gene was detected to be a plasmid for 4 out of 9 NLF *E. coli* isolates (Table 3). The genetic environment of the *bla*_CTX-M-15_ gene in most isolates (56%, 5/9) consisted of the insertion element IS*Ecp1*preceeding the locus (Table 3). The *bla*_CTX-M-15_ gene was mainly associated with the epidemiological significant clonal *E. coli* lineages such as ST131, ST167, ST1193 and ST6303. Interestingly, these strains were all NLF.

**Table 3:** Characteristics of CTX-M-15 associated NLF *E. coli* isolates.

Isolate ID	Source	ST	Phylogroup	Genome locus	MGEs^a^	Plasmid replicon
A1	Stool	6303	D	Plasmid	IS*Ecp1*	IncFIB, IncFII
A3	Stool	131	B2	Plasmid	ND^b^	Col, IncFIA, IncFIB, IncFII, IncY
A4	Stool	167	A	Chromosome	IS*Ecp1*	IncFIA, IncFIB, IncFII
A5	Stool	501	D	Chromosome	IS*Ecp1*	IncFIB, IncFII
A7	Stool	131	B2	Chromosome	ND^b^	Col, IncFIA, IncFIB, IncFII
A8	Stool	2089	A	Plasmid	ISKpn19	IncFIB
A9	Stool	1193	B2	Chromosome	IS*Ecp1*	Col, IncFIA
U-8	Urine	131	B2	Plasmid	IS3	ND^b^
U-12	Urine	131	B2	Chromosome	IS*Ecp1*	Col, IncFIA, IncFIB, IncL

a Mobile genetic elements.

b Not detected.

We screened for mutations in the quinolone-resistance-determining regions (QRDRs) of *gyrA*, *parC* and *parE*. We identified amino acid substitutions at codon positions S83I (16/17 isolates), D87N (9/17 isolates) in the amino acid sequence of *gyrA* and S80I (9/17 isolates), S57T (one isolate), E84V (4/17 isolates), E84G (two isolates) and S80R (two isolates) in *parC*. We also identified mutations at codon positions S45A, I529L and L416F in the *parE* gene. The *gyrA* S83L mutation was strongly associated with resistance to ciprofloxacin. The ESBL gene *bla*_CTX-M-15_ and *gyrA* S83L, *parC* E84V were strongly linked in ST131 and ST167 lineage strains. Additionally, the isolates also harboured acquired AMR genes encoding fluoroquinolone resistance, including *Qnrs1* (one isolate), *Qnrs13* (one isolate) and *aac(6′)-Ib-cr gene* (3 isolates). All the three acquired fluroquinolone resistance genes were associated with ciprofloxacin resistance.

MDR was frequently observed in isolates with multiple AMR genes identified in eight (47%) isolates. Cephalosporin resistance genes and the *gyrA* S83L mutation were strongly associated with the MDR phenotype. PlasmidFinder identified seven unique plasmid replicon groups, with each isolate carrying an average of 2.5 plasmid groups (Figure 4). FIB was the most commonly encountered plasmid group (*n* = 13/17), followed by FII (*n* = 9/17), CoI (*n* = 8/12) and FIA (*n* = 7/17). FIA, FIB and CoI plasmids were strongly associated with ST131 *E. coli*. The New Delhi metallo-β-lactamase (NDM)-positive *E. coli* strain (A4) concomitantly hosted three IncF-type replicons, FIA, FIB, and FII groups. We could not associate AMR genes with known plasmid replicons in two strains (A6 and U8) as no replicons were detected in them. Surprisingly, these strains belong to well-known ESBL-producing lineages (ST501 and ST131, respectively).

## Discussion

We found a high prevalence of epidemiologically significant clonal lineages amongst NLF *E. coli* isolates from patients with suspected UTIs and enteric infections in a referral diagnostic centre (icddr,b) in Dhaka, Bangladesh. None of the previous studies has focused on the genomic epidemiology of NLF *E. coli*. In the current study, we carried out a comprehensive characterisation of 17 NLF *E. coli* isolates to delineate their microbiological, biochemical, and molecular characterisation using WGS to identify their population composition and detect their molecular markers of AMR and virulence. This first report forms a baseline study on the genomic epidemiology of clinical NLF *E. coli* isolates. Knowledge of such strains’ virulence and resistance properties is important to help direct researchers, clinicians, and laboratory technologists screen, analyse and report atypical variants of *E. coli* recovered from clinical samples.

In line with previous reports (Hossain, 2012; Yaratha et al., 2017), our study revealed that the prevalence rate of NLF *E. coli* in patients in the community is around 10%. This makes it important to identify NLF *E. coli* in clinical specimens, which would help initiate appropriate antimicrobial therapy. Given the difficulty in differentiating between NLF *E. coli* and *Shigella* spp., multiple tests need to be performed as described in this study, including serology, biochemical identification (manual and API) and molecular methods like WGS. It is reported that routine MALDI-TOF MS analysis cannot reliably differentiate between *E. coli* and *Shigella* species (Ling et al., 2019). It can be noted here that the chromogenic media-CHROMagar Orientation approach cannot distinguish between lactose fermenting and NLF *E. coli* and can be used to isolate/identify both types of *E. coli* in a single step.

The predominant sequence types detected in this study were epidemiologically significant high-risk clones, including ST131, ST1193, ST12, ST73 and ST167. Our group previously reported these clones from Bangladesh amongst ESBL-producing lactose fermenting *E. coli* (Mazumder et al., 2021). None of the studies has reported them from NLF *E. coli* except for ST1193 (Wu et al., 2017). The dominance of clonal STs amongst this collection of NLF *E. coli* is an interesting finding. It warrants close attention because the inclusion of isolates in this study was not based on any AMR phenotypes and genotypes. These STs are predominantly reported to be associated with extraintestinal infections (Pitout, 2012; Riley, 2014; Manges et al., 2019). Also, a review article on global extraintestinal pathogenic *E. coli* STs enlisted these STs to be responsible for the enormous burden of extraintestinal human infections globally (Manges et al., 2019). These highly successful clonal groups are a major mode of spreading AMR by the mechanism of global expansion (Shaik et al., 2017; Tchesnokova et al., 2019).

ST131 *E. coli* is an established pandemic extraintestinal MDR pathogen (Rogers et al., 2011; Hussain et al., 2012, 2014; Nicolas-Chanoine et al., 2014). The four ST131 isolates identified in our collection were of two O and CH types, two ST131 *E. coli* exhibited O25:H4 with fumC40:fimH30 and the other two ST131 *E. coli* had O16:H5 serogroup and fumC40:fimH41 CH type. The two strains with O25:H4 serogroup belonged to the subclade C2, known as the H30Rx sub-lineage, which is the highly drug-resistant and successful sub-lineage of the ST131 clone. ST1193 is reported to be an emerging multi-resistant clonal group (Johnson et al., 2019). The three ST1193 isolates in this study carried the same set of three nonsynonymous chromosomal mutations in *gyrA* (S83L), *gyrA* (D87N) and *parC* (S80I) that confer fluoroquinolone resistance and had O75:H5 serogroup with fumC14: fimH64 CH type, they were all ciprofloxacin-resistant. ST1193 prevalence is increasing rapidly worldwide and is expected to replace the most successful clone, ST131, in the near future (Pitout et al., 2022). The three ST12 isolates in this collection were qualified as ExPEC pathogens and were ciprofloxacin-resistant. ST12 is reported to be one of the predominant STs that cause bloodstream infections (Kallonen et al., 2017; Manges et al., 2019). The ST73 isolate detected in this study harboured 104 of 335 virulence genes, including several markers of extraintestinal pathogenic and sepsis-associated *E. coli*. In contrast, it carried a smaller number of AMR genes. It demonstrated β-hemolysis on 5% sheep blood agar and had the CHtype-fumC24:fimH30. THE ST167 strain we identified showed a difficult-to-treat phenotype and carried *bla*_NDM-5_ gene. It qualified as an EAEC pathotype and this strain has been associated with a global spread of ESBL-*E. coli* in humans (Ewers et al., 2012). ST167 associated with *bla*_NDM-5_ gene was reported as an emerging carbapenem-resistant high-risk clone (Garcia-Fernandez et al., 2020).

Overall, the NLF *E. coli* isolates carried the AMR genes and were moderately resistant to antibiotics, but a higher number were assigned to different pathotypes. The isolates carried extensive virulence genes that belonged to different categories, including adhesion, invasion, colonisation, and iron uptake ability. Nevertheless, the combination of resistance and virulence in these NLF *E. coli* lineages could contribute to their increased fitness and potential to expand globally.

This study has multiple limitations. First, limited clinical and epidemiological data were available. Second, a small sample size constrained the power to generalise our findings and confirm statistical associations, particularly with the high-risk clones. The main gain of the study is that emphasis on inclusion was not laid on MDR or ESBL phenotypes; this has led to a clear understanding of the important extraintestinal pathogenic lineages within NLF *E. coli*. The WGS approach helped to provide a greater resolution to the study observations.

In conclusion, the prevalence of NLF *E. coli* variants isolated at a referral diagnostic centre (icddr,b) in Dhaka, Bangladesh, was moderate (10%). Our study revealed multi-resistant strains and high-risk clonal groups, including ST131, ST1193, ST12, ST73 and ST167, emerging within NLF *E. coli* isolates. To our knowledge, this is the first report on such a phenomenon in NLF *E. coli*; most studies focus on pathotypes encompassing single STs. These high-risk clones may further evolve by acquiring carbapenem and colistin resistance genes to cause difficult-to-treat infections. Therefore, strengthening microbiology laboratories to detect and report NLF *E. coli* isolates is important to accomplish successful patient treatment and to feed data to AMR surveillance programs. Further national and regional multicentre “one health” studies are required to ascertain the significance of NLF *E. coli* as significant pathogens impacting public health.

## Data availability statement

The datasets presented in this study can be found in online repositories. The names of the repository/repositories and accession number(s) can be found in the article.

## Author contributions

RM and AH designed the study and drafted the manuscript. RM performed genome sequencing. AM, AH, and RM carried out the bioinformatics analyses and interpretation of results and prepared tables and figures. RM and SH performed microbiological tests. JP, SC, MA, DA, TC, and DM contributed to the discussions and reviewed the manuscript. DM supervised the study. All authors contributed to the article and approved the submitted version.

## Conflict of interest

The authors declare that the research was conducted in the absence of any commercial or financial relationships that could be construed as a potential conflict of interest.

## Publisher’s note

All claims expressed in this article are solely those of the authors and do not necessarily represent those of their affiliated organizations, or those of the publisher, the editors and the reviewers. Any product that may be evaluated in this article, or claim that may be made by its manufacturer, is not guaranteed or endorsed by the publisher.

## References

[ref1] Aditi F. Y. Rahman S. S. Hossain M. M. (2017). A study on the microbiological status of mineral drinking water. Open Microbiol. J. 11, 31–44. doi: 10.2174/1874285801711010031, PMID: 28603564 PMC5447907

[ref2] Baddam R. Sarker N. Ahmed D. Mazumder R. Abdullah A. Morshed R.. (2020). Genome dynamics of vibrio cholerae isolates linked to seasonal outbreaks of cholera in Dhaka, Bangladesh. MBio 11:e03339-19. doi: 10.1128/mBio.03339-19, PMID: 32047137 PMC7018647

[ref3] Bankevich A. Nurk S. Antipov D. Gurevich A. A. Dvorkin M. Kulikov A. S.. (2012). SPAdes: A new genome assembly algorithm and its applications to single-cell sequencing. J. Comput. Biol. 19, 455–477. doi: 10.1089/cmb.2012.0021, PMID: 22506599 PMC3342519

[ref4] Beghain J. Bridier-Nahmias A. Le Nagard H. Denamur E. Clermont O. (2018). Clermon typing: an easy-to-use and accurate in silico method for Escherichia genus strain phylotyping. Microb. Genomics 4:192. doi: 10.1099/mgen.0.000192, PMID: 29916797 PMC6113867

[ref5] Bertels F. Silander O. K. Pachkov M. Rainey P. B. Van Nimwegen E. (2014). Automated reconstruction of whole-genome phylogenies from short-sequence reads. Mol. Biol. Evol. 31, 1077–1088. doi: 10.1093/MOLBEV/MSU088, PMID: 24600054 PMC3995342

[ref6] Bopp C. A. Brenner F. W. Fields P. I. Wells J. G., (2003). Manual of Clinical Microbiology. 8th *Edn.*. Washington, DC: ASM Press.

[ref7] Bortolaia V. Kaas R. S. Ruppe E. Roberts M. C. Schwarz S. Cattoir V.. (2020). Res finder 4.0 for predictions of phenotypes from genotypes. J. Antimicrob. Chemother. 75, 3491–3500. doi: 10.1093/jac/dkaa345, PMID: 32780112 PMC7662176

[ref8] Brooks K. Sodeman T. (1974). A rapid method for determining decarboxylase and dihydrolase activity. J. Clin. Pathol. 27, 148–152. doi: 10.1136/JCP.27.2.148, PMID: 4824992 PMC478029

[ref9] Carattoli A. Zankari E. García-Fernández A. Voldby Larsen M. Lund O. Villa L.. (2014). *In Silico* detection and typing of plasmids using plasmid finder and plasmid multilocus sequence typing. Antimicrob. Agents Chemother. 58, 3895–3903. doi: 10.1128/AAC.02412-14, PMID: 24777092 PMC4068535

[ref10] Chakraborty A. Adhikari P. Shenoy S. Saralaya V. (2016). Virulence factor profiles, phylogenetic background, and antimicrobial resistance pattern of lactose fermenting and nonlactose fermenting *Escherichia coli* from extraintestinal sources. Indian J. Pathol. Microbiol. 59, 180–184. doi: 10.4103/0377-4929.182032, PMID: 27166036

[ref11] Chang J. Yu J. Lee H. Ryu H. Park K. Park Y. J. (2014). Prevalence and characteristics of lactose non-fermenting *Escherichia coli* in urinary isolates. J. Infect. Chemother. 20, 738–740. doi: 10.1016/J.JIAC.2014.07.005, PMID: 25193040

[ref12] Chen L. Yang J. Yu J. Yao Z. Sun L. Shen Y.. (2005). VFDB: A reference database for bacterial virulence factors. Nucleic Acids Res. 33, D325–D328. doi: 10.1093/nar/gki008, PMID: 15608208 PMC539962

[ref13] Chen S. Zhou Y. Chen Y. Gu J. (2018). Fastp: an ultra-fast all-in-one FASTQ preprocessor. Bioinformatics 34, i884–i890. doi: 10.1093/bioinformatics/bty560, PMID: 30423086 PMC6129281

[ref14] Clausen P. T. L. C. Aarestrup F. M. Lund O. (2018). Rapid and precise alignment of raw reads against redundant databases with KMA. BMC Bioinformatics 19, 1–8. doi: 10.1186/S12859-018-2336-6/TABLES/2 30157759 PMC6116485

[ref15] Colonna B. Ranucci L. Fradiani P. A. Casalino M. Calconi A. Nicoletti M. (1992). Organisation of aerobactin, hemolysin, and antibacterial resistance genes in lactose-negative *Escherichia coli* strains of serotype O4 isolated from children with diarrhea. Infect. Immun. 60, 5224–5231. doi: 10.1128/IAI.60.12.5224-5231.1992, PMID: 1452355 PMC258301

[ref16] Eren A. M. Esen O. C. Quince C. Vineis J. H. Morrison H. G. Sogin M. L.. (2015). Anvi’o: an advanced analysis and visualisation platformfor’omics data. Peer J 2015:1319. doi: 10.7717/PEERJ.1319/SUPP-5 PMC4614810 26500826

[ref17] Ewers C. Bethe A. Semmler T. Guenther S. Wieler L. H. (2012). Extended-spectrum β-lactamase-producing and AmpC-producing *Escherichia coli* from livestock and companion animals, and their putative impact on public health: a global perspective. Clin. Microbiol. Infect. 18, 646–655. doi: 10.1111/J.1469-0691.2012.03850.X, PMID: 22519858

[ref18] Flores-Mireles A. L. Walker J. N. Caparon M. Hultgren S. J. (2015). Urinary tract infections: epidemiology, mechanisms of infection and treatment options. Nat. Rev. Microbiol. 13, 269–284. doi: 10.1038/nrmicro3432, PMID: 25853778 PMC4457377

[ref19] Garcia-Fernandez A. Villa L. Bibbolino G. Bressan A. Trancassini M. Pietropaolo V.. (2020). Novel insights and features of the NDM-5-producing *Escherichia coli* sequence type 167 high-risk clone. mSphere 5:e00269-20. doi: 10.1128/MSPHERE.00269-20/ASSET/6E7B35EC-775E-40A6-B00C-FDACD968538C/ASSETS/GRAPHIC/MSPHERE.00269-20-F0003.JPEG, PMID: 32350092 PMC7193042

[ref20] Guindon S. Dufayard J. F. Lefort V. Anisimova M. Hordijk W. Gascuel O. (2010). New algorithms and methods to estimate maximum-likelihood phylogenies: assessing the performance of PhyML 3.0. Syst. Biol. 59, 307–321. doi: 10.1093/SYSBIO/SYQ010, PMID: 20525638

[ref21] Gurevich A. Saveliev V. Vyahhi N. Tesler G. (2013). QUAST: quality assessment tool for genome assemblies. Bioinformatics 29, 1072–1075. doi: 10.1093/BIOINFORMATICS/BTT086, PMID: 23422339 PMC3624806

[ref22] Hasman H. Saputra D. Sicheritz-Ponten T. Lund O. Svendsen C. A. Frimodt-Moller N.. (2014). Rapid whole-genome sequencing for detection and characterisation of microorganisms directly from clinical samples. J. Clin. Microbiol. 52, 139–146. doi: 10.1128/JCM.02452-13, PMID: 24172157 PMC3911411

[ref23] Hawkey P. M. (2006). Identification of Enterobacteriaceae. 3rd *Edn.* Minneapolis: Burgess Publishing Co.

[ref24] Hossain A. (2012). Presence and pattern of virulence genes in non-lactose fermenting *Escherichia coli* strains isolated from stools of children <5 years in rural and urban Bangladesh. Int. J. Infect. Dis. 16:e395. doi: 10.1016/J.IJID.2012.05.525

[ref250] Huang J. Zhao Z. Zhang Q. Zhang S. Zhang S. Chen M.. (2021). Phylogenetic Analysis Reveals Distinct Evolutionary Trajectories of the Fluoroquinolones-Resistant *Escherichia coli* ST1193 From Fuzhou, China. Front. Microbiol. 12:3370. doi: 10.3389/FMICB.2021.746995/BIBTEX, PMID: 34803966 PMC8602892

[ref25] Hussain A. Ewers C. Nandanwar N. Guenther S. Jadhav S. Wieler L. H.. (2012). Multi-resistant uropathogenic *Escherichia coli* from a region in India where urinary tract infections are endemic: genotypic and phenotypic characteristics of sequence type 131 isolates of the CTX-M-15 extended-spectrum-β-lactamase-producing lineage. Antimicrob. Agents Chemother. 56, 6358–6365. doi: 10.1128/AAC.01099-12, PMID: 23045357 PMC3497203

[ref26] Hussain A. Ranjan A. Nandanwar N. Babbar A. Jadhav S. Ahmed N. (2014). Genotypic and phenotypic profiles of *Escherichia coli* isolates belonging to clinical sequence type 131 (ST131), clinical non-ST131, and fecal non-ST131 lineages from India. Antimicrob. Agents Chemother. 58, 7240–7249. doi: 10.1128/AAC.03320-14, PMID: 25246402 PMC4249578

[ref27] Joensen K. G. Tetzschner A. M. M. Iguchi A. Aarestrup F. M. Scheutz F. (2015). Rapid and easy in silico serotyping of *Escherichia coli* isolates by use of whole-genome sequencing data. J. Clin. Microbiol. 53, 2410–2426. doi: 10.1128/JCM.00008-15 25972421 PMC4508402

[ref28] Johansson M. H. K. Bortolaia V. Tansirichaiya S. Aarestrup F. M. Roberts A. P. Petersen T. N. (2021). Detection of mobile genetic elements associated with antibiotic resistance in salmonella enterica using a newly developed web tool: MobileElementFinder. J. Antimicrob. Chemother. 76, 101–109. doi: 10.1093/JAC/DKAA390, PMID: 33009809 PMC7729385

[ref29] Johnson T. J. Elnekave E. Miller E. A. Munoz-Aguayo J. Figueroa C. F. Johnston B.. (2019). Phylogenomic analysis of Extraintestinal pathogenic *Escherichia coli* sequence type 1193, an emerging multidrug-resistant clonal group. Antimicrob. Agents Chemother. 63:e01913-18. doi: 10.1128/AAC.01913-18, PMID: 30348668 PMC6325179

[ref30] Johnson J. R. Stell A. L. (2000). Extended virulence genotypes of *Escherichia coli* strains from patients with urosepsis in relation to phylogeny and host compromise. J. Infect. Dis. 181, 261–272. doi: 10.1086/315217, PMID: 10608775

[ref31] Kallonen T. Brodrick H. J. Harris S. R. Corander J. Brown N. M. Martin V.. (2017). Systematic longitudinal survey of invasive *Escherichia coli* in England demonstrates a stable population structure only transiently disturbed by the emergence of ST131. Genome Res. 27, 1437–1449. doi: 10.1101/GR.216606.116/-/DC1, PMID: 28720578 PMC5538559

[ref32] Kaper J. B. Nataro J. P. Mobley H. L. T. (2004). Pathogenic *Escherichia coli*. Nat. Rev. Microbiol. 2, 123–140. doi: 10.1038/nrmicro818 15040260

[ref33] Larsen M. V. Cosentino S. Lukjancenko O. Saputra D. Rasmussen S. Hasman H.. (2014). Benchmarking of methods for genomic taxonomy. J. Clin. Microbiol. 52, 1529–1539. doi: 10.1128/JCM.02981-13/SUPPL_FILE/ZJM999093347SO4.PDF, PMID: 24574292 PMC3993634

[ref34] Larsen M. V. Cosentino S. Rasmussen S. Friis C. Hasman H. Marvig R. L.. (2012). Multilocus sequence typing of total-genome-sequenced bacteria. J. Clin. Microbiol. 50, 1355–1361. doi: 10.1128/JCM.06094-11, PMID: 22238442 PMC3318499

[ref35] Letunic I. Bork P. (2016). Interactive tree of life (iTOL) v3: an online tool for the display and annotation of phylogenetic and other trees. Nucleic Acids Res. 44, W242–W245. doi: 10.1093/nar/gkw290, PMID: 27095192 PMC4987883

[ref36] Ling J. Wang H. Li G. Feng Z. Song Y. Wang P.. (2019). A novel short-term high-lactose culture approach combined with a matrix-assisted laser desorption ionisation-time of flight mass spectrometry assay for differentiating *Escherichia coli* and *Shigella* species using artificial neural networks. PLoS One 14:e0222636. doi: 10.1371/JOURNAL.PONE.0222636, PMID: 31593573 PMC6782097

[ref37] Logan L. K. Weinstein R. A. (2017). The epidemiology of carbapenem-resistant enterobacteriaceae: the impact and evolution of a global menace 215, S28–S36. doi: 10.1093/infdis/jiw282, PMID: 28375512 PMC5853342

[ref38] Magiorakos A. P. Srinivasan A. Carey R. B. Carmeli Y. Falagas M. E. Giske C. G.. (2012). Multidrug-resistant, extensively drug-resistant and pandrug-resistant bacteria: an international expert proposal for interim standard definitions for acquired resistance. Clin. Microbiol. Infect. 18, 268–281. doi: 10.1111/J.1469-0691.2011.03570.X, PMID: 21793988

[ref39] Manges A. R. Geum H. M. Guo A. Edens T. J. Fibke C. D. Pitout J. D. D. (2019). Global extraintestinal pathogenic *Escherichia coli* (Expec) lineages. Clin. Microbiol. Rev. 32:e00135-18. doi: 10.1128/CMR.00135-18, PMID: 31189557 PMC6589867

[ref40] Mazumder R. Abdullah A. Ahmed D. Hussain A. (2020a). High prevalence of blactx-m-15 gene among extended-spectrum β-lactamase-producing *Escherichia coli* isolates causing extraintestinal infections in Bangladesh. Antibiotics. 9:796. doi: 10.3390/antibiotics9110796, PMID: 33187055 PMC7696227

[ref41] Mazumder R. Abdullah A. Hussain A. Ahmed D. Mondal D. (2020b). Draft genome sequence of Chromobacterium violaceum RDN09, isolated from a patient with a wound infection in Bangladesh. Microbiol. Resour. Announc. 9:e00957-20. doi: 10.1128/mra.00957-20, PMID: 33060272 PMC7561691

[ref42] Mazumder R. Hussain A. Abdullah A. Islam M. N. Sadique M. T. Muniruzzaman S. M.. (2021). International high-risk clones among extended-Spectrum β-lactamase–producing *Escherichia coli* in Dhaka, Bangladesh. Front. Microbiol. 12:2843. doi: 10.3389/FMICB.2021.736464/BIBTEX PMC8521144 34671331

[ref43] Nataro J. P. Kaper J. B. (1998). Diarrheagenic *Escherichia coli*. Clin. Microbiol. Rev. 11, 142–201. doi: 10.1128/CMR.11.1.142/FORMAT/EPUB, PMID: 9457432 PMC121379

[ref44] Nia Santos Braz V. Melchior K. Gallina Moreira C. (2020). *Escherichia coli* as a multifaceted pathogenic and versatile bacterium. Front. Cell. Infect. Microbiol. 10:548492. doi: 10.3389/fcimb.2020.548492, PMID: 33409157 PMC7779793

[ref45] Nicolas-Chanoine M.-H. Bertrand X. Madec J.-Y. (2014). *Escherichia coli* ST131, an intriguing clonal group. Clin. Microbiol. Rev. 27, 543–574. doi: 10.1128/CMR.00125-13, PMID: 24982321 PMC4135899

[ref46] Nicoletti M. Superti F. Conti C. Calconi A. Zagaglia C. (1988). Virulence factors of lactose-negative *Escherichia coli* strains isolated from children with diarrhea in Somalia. J. Clin. Microbiol. 26, 524–529. doi: 10.1128/JCM.26.3.524-529.1988, PMID: 3281977 PMC266325

[ref47] Nusrat N. Ridwan B. R. Anowara B. Humaira A. (2017). Isolation, identification and molecular characterisation of rhizobium species from Sesbania bispinosa cultivated in Bangladesh. Afr. J. Agric. Res. 12, 1874–1880. doi: 10.5897/AJAR2017.12321

[ref48] Pitout J. D. D. (2012). Extraintestinal pathogenic *Escherichia coli*: a combination of virulence with antibiotic resistance. Front. Microbiol. 3:9. doi: 10.3389/fmicb.2012.00009, PMID: 22294983 PMC3261549

[ref49] Pitout J. D. D. Peirano G. Chen L. DeVinney R. Matsumura Y. (2022). *Escherichia coli* ST1193: following in the footsteps of *E. coli* ST131. Antimicrob. Agents Chemother. 66:e0051122. doi: 10.1128/AAC.00511-22, PMID: 35658504 PMC9295538

[ref50] Riley L. W. (2014). Pandemic lineages of extraintestinal pathogenic *Escherichia coli*. Clin. Microbiol. Infect. 20, 380–390. doi: 10.1111/1469-0691.12646, PMID: 24766445

[ref51] Roer L. Johannesen T. B. Hansen F. Stegger M. Tchesnokova V. Sokurenko E.. (2018). CHTyper, a web tool for subtyping of Extraintestinal pathogenic *Escherichia coli* based on the fumC and fimH alleles. J. Clin. Microbiol. 56, 63–81. doi: 10.1128/JCM.00063-18, PMID: 29436420 PMC5869820

[ref52] Rogers B. A. Sidjabat H. E. Paterson D. L. (2011). *Escherichia coli* O25b-ST131: a pandemic, multi-resistant, community-associated strain. J. Antimicrob. Chemother. 66, 1–14. doi: 10.1093/JAC/DKQ415, PMID: 21081548

[ref53] Shaik S. Ranjan A. Tiwari S. K. Hussain A. Nandanwar N. Kumar N.. (2017). Comparative genomic analysis of globally dominant ST131 clone with other epidemiologically successful Extraintestinal pathogenic *Escherichia coli* (ExPEC) lineages. MBio 8:e01596-17. doi: 10.1128/mBio.01596-17, PMID: 29066550 PMC5654935

[ref54] Siqueira F. M. De Carli S. Lopes C. E. Machado L. Vieira T. R. Pöppl G.. (2021). Non-lactose-fermenting uropathogenic *Escherichia coli* from dogs: virulence profile characterization. Lett. Appl. Microbiol. 72, 596–603. doi: 10.1111/LAM.13454, PMID: 33524173

[ref55] Tatusova T. Dicuccio M. Badretdin A. Chetvernin V. Nawrocki E. P. Zaslavsky L.. (2016). NCBI prokaryotic genome annotation pipeline. Nucleic Acids Res. 44, 6614–6624. doi: 10.1093/NAR/GKW569, PMID: 27342282 PMC5001611

[ref56] Tchesnokova V. Radey M. Chattopadhyay S. Larson L. Lee Weaver J. Kisiela D.. (2019). Pandemic fluoroquinolone resistant *Escherichia coli* clone ST1193 emerged via simultaneous homologous recombinations in 11 gene loci. Proc. Natl. Acad. Sci. U. S. A. 116, 14740–14748. doi: 10.1073/pnas.1903002116 31262826 PMC6642405

[ref57] Wu J. Lan F. Lu Y. He Q. Li B. (2017). Molecular characteristics of ST1193 clone among phylogenetic group B2 non-ST131 fluoroquinolone-resistant *Escherichia coli*. Front. Microbiol. 8:2294. doi: 10.3389/FMICB.2017.02294/BIBTEX, PMID: 29209300 PMC5702334

[ref58] Yaratha G. Perloff S. Changala K. (2017). Lactose vs non-lactose fermenting *E. coli*: epidemiology, clinical outcomes, and resistance. Open Forum Infect. Dis. 4, S589–S590. doi: 10.1093/OFID/OFX163.1546

[ref59] Zankari E. Allesøe R. Joensen K. G. Cavaco L. M. Lund O. Aarestrup F. M. (2017). PointFinder: A novel web tool for WGS-based detection of antimicrobial resistance associated with chromosomal point mutations in bacterial pathogens. J. Antimicrob. Chemother. 72, 2764–2768. doi: 10.1093/jac/dkx217, PMID: 29091202 PMC5890747

